# Electrospun NiO/carbon nanofiber hole transport layers for perovskite solar cell performance

**DOI:** 10.55730/1300-0527.3753

**Published:** 2025-08-05

**Authors:** Thanh Tung PHAM, Dan Thuy VAN PHAM, Viet Nhan Hoa NGUYEN, Van Hong Thien DOAN

**Affiliations:** 1 Department of Energy and Chemical Engineering, Incheon National University, Incheon, Korea; 2 Faculty of Chemical Engineering, College of Engineering, Can Tho University, Can Tho City, Viet Nam

**Keywords:** Carbon, electrospinning, hole layer, nickel(II) oxide, perovskite solar cells

## Abstract

Rapid technological advancement has increased energy consumption, environmental pollution, and climate change, necessitating sustainable sources of energy. Solar energy is appealing because it is renewable and has minimal environmental impact. However, the complex fabrication and high costs of conventional silicon solar cells have driven research into alternative technologies like perovskite solar cells (PSCs). This study investigates the use of electrospun nickel oxide (NiO)/carbon nanofibers as efficient hole-transporting layers in PSCs. NiO nanomaterials were synthesized via coprecipitation and integrated into polyvinyl alcohol nanofibers through electrospinning at an optimized concentration of 20 g/L, producing uniform nanofibers with mean (SD) diameter of 136 nm (20). The electrospun nanofibers were calcined to convert them into NiO/carbon nanofibers. The structural and chemical properties of nanofibers were characterized using scanning electron microscopy, transmission electron microscopy, X-ray diffraction, Fourier transform infrared (FTIR) and Raman spectroscopy, thermogravimetric analysis, and photoluminescence spectroscopy. These NiO-based nanofibers were used as hole-conducting layers in constructing fluorine-doped tin oxide (FTO)/titanium dioxide peroxide P25 (TiO_2_)/perovskite/NiO-carbon/FTO solar cells, achieving a maximum efficiency of 5.96%. This research shows the potential of NiO-based hole-conducting layers in enhancing PSC efficiency. Optimizing NiO concentration and electrospinning time can significantly improve the conductive properties and performance of PSCs.

## 1. Introduction

The rapid advancement of science and technology has significantly escalated energy consumption, environmental pollution, and climate change. Consequently, the pursuit of green, clean, and sustainable energy sources has emerged as a critical contemporary challenge [1–3]. Among these sources, solar energy stands out due to its renewable nature and minimal environmental impact during extraction [1–5]. Recent improvements in solar cell manufacturing have enhanced productivity and design flexibility, particularly through innovations like crystalline silicon wafers [6–8]. However, conventional silicon solar cells have moderate efficiencies of approximately 20% and require complex fabrication processes, prompting the exploration of alternative technologies [2,9].

Since their introduction in 2013, perovskite solar cells (PSCs) have garnered attention for their promising attributes, including high efficiency surpassing 25% [2,10–15]. Key improvements have focused on enhancing the durability of the perovskite layer and exploring novel materials for electrodes [12–14,16,17]. Notably, nanotechnology has enabled significant improvements in optimizing materials and processes, particularly in the development of hole conductor layers crucial for PSCs.

Nickel oxide (NiO) nanomaterials have been extensively researched for their chemical, structural, optical, magnetic, and electrical properties. Various synthesis methods, such as coprecipitation, sol-gel, and hydrothermal processes, have proven effective in controlling size and material characteristics [10,11,18–20]. NiO, often doped with metals like Cu, Sn, Pb, or Co, has favorable attributes such as suitable bandgap position, high stability, and efficient hole conduction [21,22]. Recent studies have shown the efficacy of NiO in achieving solar cell efficiency nearing 18%, particularly when used in conjunction with novel fabrication techniques like electrospinning and radio frequency magnetron sputtering [23–25]. To enhance the stability of PSCs, incorporating hole-conducting materials such as NiO and carbon is an effective approach [16,17,22]. Low-cost carbon materials enhance the stability and charge transfer efficiency of PSCs. Due to its appropriate work function of 5.0 eV, carbon is an optimal choice for the back electrode of PSCs [21,26,27]. Consequently, carbon-based PSCs have been developed to fulfill the demand for both low cost and high stability.

Further innovations include the use of polymeric materials for creating efficient hole layers via electrospinning technology [28–31]. Polyvinyl alcohol (PVA) is an effective precursor for nanofiber fabrication due to its excellent electrospinning ability, enabling the formation of uniform PVA/NiO nanofibers [3,32]. These nanofibers can be subsequently carbonized to produce NiO/carbon nanofibers that serve as the hole transport layer in PSCs. The carbonization process enhances electrical conductivity while preserving the NiO distribution within the fiber matrix, improving charge transport properties. This approach enables precise control over the NiO content and structural characteristics, facilitating detailed analysis and optimization of solar cell models.

This study investigated the use of electrospun NiO/carbon nanofibers as efficient hole-transporting layers in PSCs. NiO nanoparticles were synthesized using a coprecipitation methods and subsequently incorporated into PVA nanofibers via electrospinning. Through calcination, these nanofibers were converted into NiO/carbon nanofibers that were used as a hole-conducting layer in PSCs. The investigation primarily focuses on optimizing the composition of NiO within the PVA fibers to maximize the solar cell conversion efficiency.

## 2. Materials and methods

### 2.1. Materials

Nickel chloride hexahydrate (NiCl_2_.6H_2_O), sodium hydroxide (NaOH), PVA, methyl amine (CH_3_NH_2_), hydroiodic acid (HI), lead iodide (PbI_2_), and dimethylformamide (DMF) were purchased from Sigma-Aldrich (St. Louis, MO, USA). Titanium dioxide peroxide P25 (TiO_2_) was obtained from Thermo Fisher Scientific Chemicals (Waltham, MA, USA). Fluorine-doped tin oxide (FTO) glasses were provided by Well Join Industry Co., Ltd.

### 2.2. Methods

#### 2.2.1 Preparation of NiO/carbon nanofibers

NiO nanoparticles were synthesized via the coprecipitation method [33]. Initially, 2.43 g of NiCl_2_.6H_2_O was dissolved in 40 mL of deionized water and stirred at room temperature for 10 min until a homogeneous dark green solution formed. A 5 M NaOH solution was slowly added until the pH reached 10. After stirring for an additional 10 min, the precipitate was washed 3 times with deionized water (pH 7) and centrifuged. The precipitate was dried at 100 °C for 5 h followed by calcination at 400 °C for 2 h to yield NiO nanoparticles.

A specific quantity of NiO nanoparticles (ranging from 30 to 50 mg) was dispersed in 2 mL of distilled water under ultrasonication at 40 kHz and 30 °C for 10 min. Subsequently, 220 mg of PVA was added to the suspension and stirred at 80 °C for 2 h to obtain a NiO/PVA suspension. The suspension was cooled to room temperature prior to electrospinning. The homogeneous NiO/PVA suspension was loaded into a syringe equipped with a metallic needle for electrospinning. The metallic needle was connected to a high-voltage power source (15 kV), and the suspension was electrospun onto FTO glass substrates positioned 15 cm away from the needle tip. The resulting nanofibers were calcined at 500 °C for 2 h.

#### 2.2.2 Characteristics of NiO/carbon nanofibers

The morphology of NiO/carbon nanofibers was examined using scanning electron microscopy (SEM) (Hitachi S4800-NIHE, Tokyo, Japan) at 10 kV after gold coating, and transmission electron microscopy (TEM) (JEOL JEM-1400, Tokyo, Japan). The crystal structures were analyzed by X-ray diffraction (XRD) (D8 Advance, Bruker, Billerica, MA, USA) from 10° to 80° (2θ) using CuKα radiation. Raman spectroscopy (LabRAM HR 800, Horiba Scientific, Kyoto, Japan) and Fourier transform infrared spectroscopy (FTIR) (Nicolet 6700, Thermo Fisher Scientific) were used to study the functional groups over the range of 4000–400 cm^−1^. Thermogravimetric analysis (TGA) (NETZSCH, Selb, Germany) was used to investigate the phase change of PVA and NiO content in NiO/PVA nanofibers by heating samples from 30 to 800 °C at 10 °C/min under a nitrogen flow rate of 10 mL/min. Photoluminescence (PL) spectra were measured using a Fluorolog-3 fluorescence measurement system (Horiba Scientific).

#### 2.2.3 Preparation of CH_3_NH_3_PbI_3_ precursor solution

CH_3_NH_3_I precursor was synthesized by reacting methyl amine (CH_3_NH_2_) with hydroiodic acid (HI) in a 1:1 molar ratio at 0 °C for 2 h. The resulting solution was dried at 80 °C for 4 h, washed 3 times with diethyl ether, and further dried at 130 °C for 30 min. The perovskite precursor solution was then prepared by mixing CH_3_NH_3_I (0.159 g) and PbI_2_ (0.461 g) in 1 mL of DMF, stirring at 60 °C for 30 min to form a bright yellow transparent solution [30,34].

#### 2.2.4 Solar cell fabrication and testing

FTO glasses were sequentially cleaned with deionized water, methanol, acetone, and isopropanol, each for 10 min at 30 °C followed by sonication at 40 kHz. The glasses were dried at 150 °C for 10 min. The NiO/PVA nanofibers were electrospun onto the FTO glass at a concentration of 20 g/L and varied machining times (10, 20, and 30 min). The glasses were calcined in a nitrogen atmosphere at 500 °C for 2 h and cooled slowly for 5 h for complete carbonization of PVA.

Next, the perovskite precursor layer was spin-coated at 2000 rpm for 60 s onto the NiO/carbon nanofiber layer and annealed at 100 °C for 30 min to form the perovskite layer. The TiO_2_ electron-conducting layer was fabricated using a 20 g/L TiO_2_ nanoparticle solution in ethanol that was spin-coated onto the perovskite layer at 2000 rpm for 60 s, and annealed at 100 °C for 30 min. Finally, another layer of FTO glass completed the solar cell structure. All processes were conducted in a nitrogen-filled glovebox.

The solar cell panels were structured as follows:

1. FTO/(nano TiO_2_)/perovskite/NiO-carbon (10 min electrospinning)/FTO
2. FTO/(nano TiO_2_)/perovskite/NiO-carbon (20 min electrospinning)/FTO
3. FTO/(nano TiO_2_)/perovskite/NiO-carbon (30 min electrospinning)/FTO

**Figure 1:**
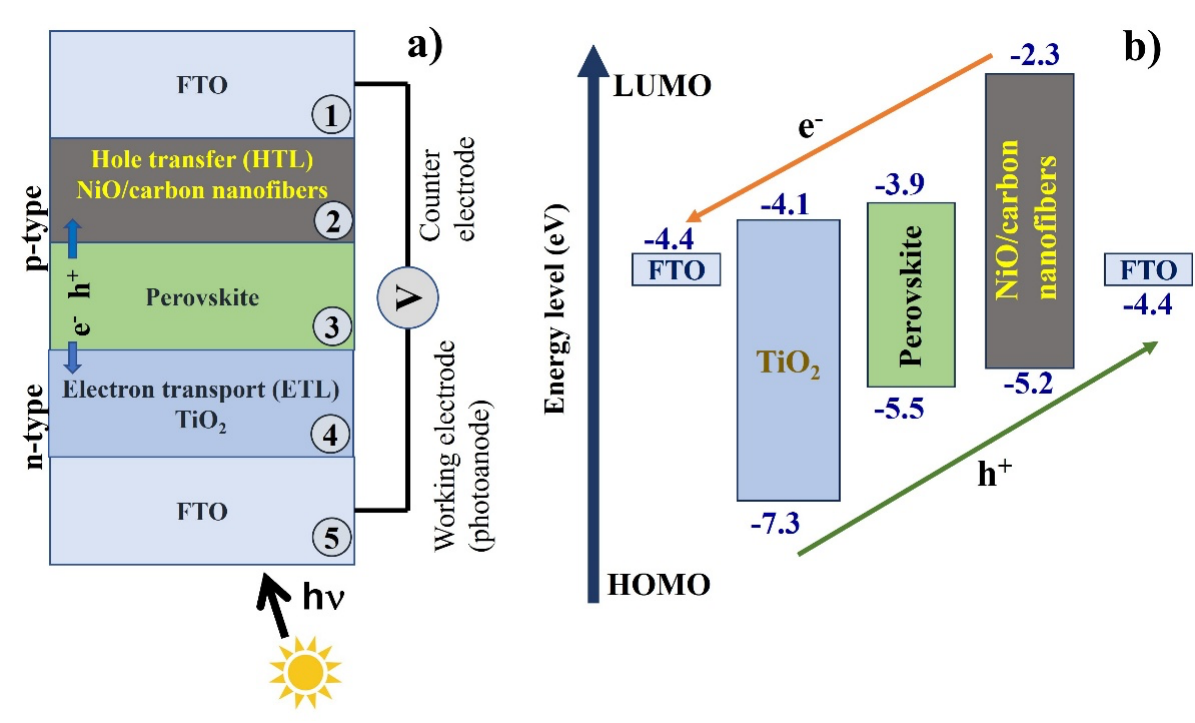
(a) Schematic diagram of the perovskite solar cell structure. (b) Energy band alignment of each functional layer.

A Keithley source meter unit (model 2450) (Solon, OH, USA) and a SAN-EI electric solar simulator (model XES-40S3) (Chiba, Japan) with an active area of 0.9 cm^2^ were used to obtain photoelectric measurements of the solar cell panels. These measurements were used to construct J–V curves. Figure 1a presents the schematic structure of a perovskite solar cell, consisting of FTO and TiO_2_ as the electron transport layer (ETL), a perovskite light-absorbing layer, and NiO/carbon nanofibers serving as the hole transport layer (HTL). Figure 1b illustrates the energy level alignment of each component. Efficient charge transfer from the perovskite layer to the ETL and HTL was facilitated by favorable energy band alignment.

## 3. Results and discussion

### 3.1. Preparation of NiO/carbon nanofibers

#### 3.1.1 Effect of amount of NiO on the electrospinning process

**Figure 2:**
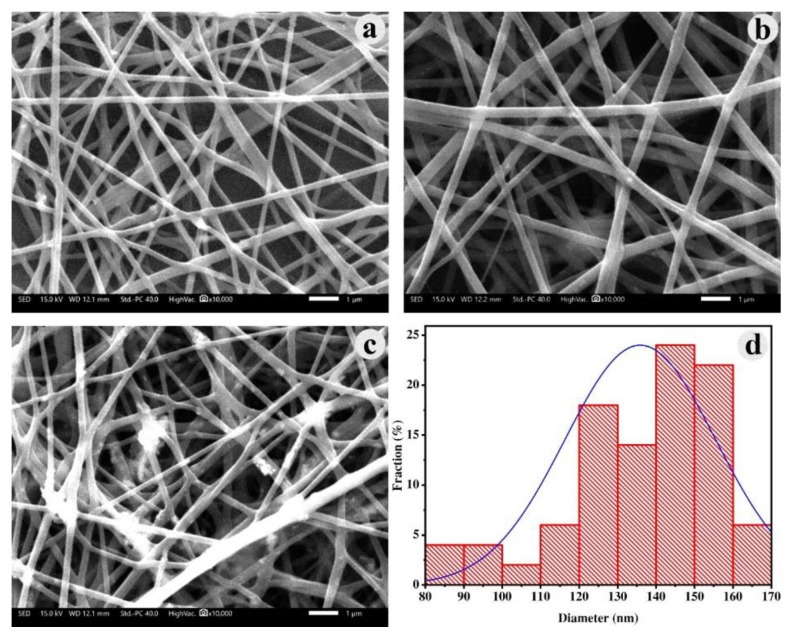
SEM images of NiO/PVA nanofibers with various NiO concentrations: (a) 15 g/L, (b) 20 g/L, (c) 25 g/L, and (d) particle size distribution of NiO/PVA nanofibers at 20 g/L.

Figure 2 displays SEM images of NiO/PVA nanofibers fabricated through electrospinning. The electrospun NiO/PVA nanofibers had relatively consistent diameters across different concentrations. Samples with NiO concentrations of 15 and 20 g/L had well-dispersed NiO nanoparticles within the fibers, resulting in a uniform fiber morphology with minimal granulation during spinning (Figures 2a and 2b). Conversely, at a concentration of 25 g/L, the dispersion of NiO nanoparticles was less effective, leading to fibers with noticeable agglomeration (Figure 2c). Thus, a concentration of 20 g/L of NiO was optimal for forming the hole-conducting layer in PSCs. The nanofiber diameters postspinning predominantly ranged from 90 to 170 nm, with an average size of 136 nm and a standard deviation of 20 nm (Figure 2d).

#### 3.1.2 Characteristics of NiO/carbon nanofibers

##### Morphological analysis of NiO/carbon nanofibers by SEM

**Figure 3:**
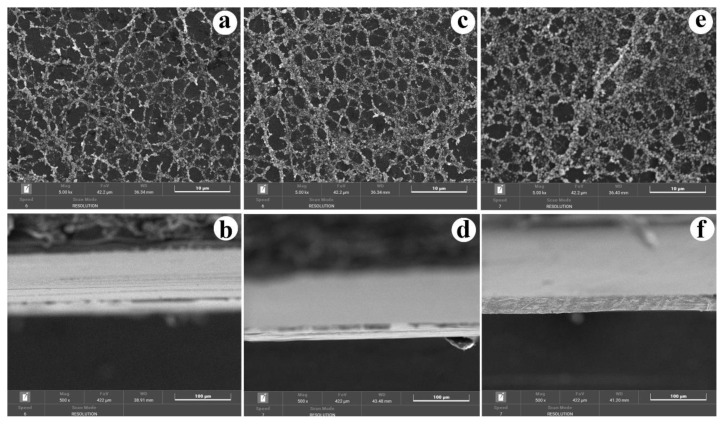
SEM images of NiO/carbon nanofibers at 10 min (a and b), 20 min (c and d), and 30 min (e and f). Surface (a, c, and e) and cross-section (b, d, and f).

Figure 3 presents the SEM images of NiO/carbon nanofibers electrospun for different durations: 10 min (Figures 3a and 3b), 20 min (Figure 3c and 3d), and 30 min (Figure 3e and 3f). After calcination, the morphology of the original NiO/PVA nanofibers underwent a notable transformation due to the thermal conversion of PVA into carbon. The resulting NiO/carbon nanofibers had a relatively uniform structure, consisting of interconnected nanoparticles forming continuous fibrous networks. A reduction in fiber diameter was observed after calcination, attributed to the decomposition of PVA and the densification of the carbon matrix. Furthermore, as the electrospinning duration increased from 10 to 30 min, a clear increase in the amount of deposited fibers was noted, resulting in thicker nanofiber mats. This trend was confirmed by the cross-sectional SEM images (Figures 3b, 3d, and 3f), where the thickness of the nanofiber layers gradually increased with longer spinning time. These results indicate that electrospinning time plays a crucial role in controlling the fiber mat thickness and overall yield, while calcination ensures structural transformation and stabilization of the NiO/carbon nanofiber network.

##### XRD

**Figure 4:**
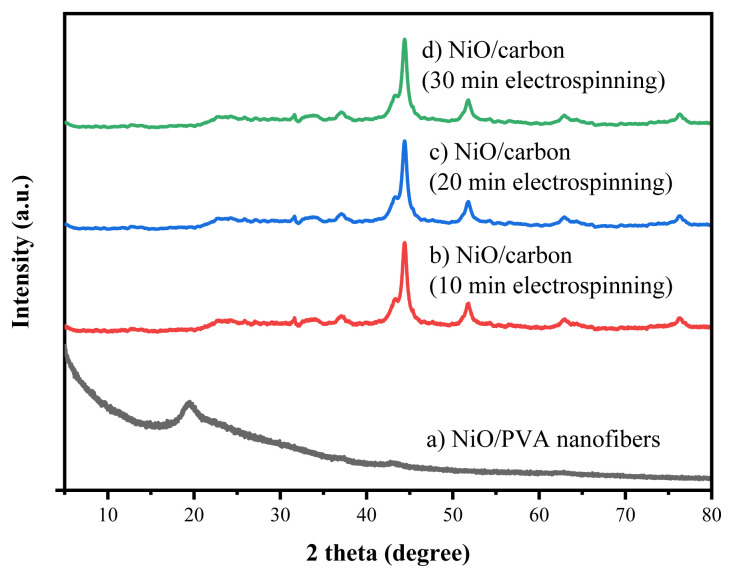
XRD patterns of (a) NiO/PVA nanofibers and NiO/carbon nanofibers electrospun for (b) 10 min, (c) 20 min, and (d) 30 min.

Figure 4 shows the XRD patterns of NiO/PVA nanofibers before and after calcination at 500 °C for 2 h. The diffraction pattern of the as-spun NiO/PVA nanofibers displays a broad peak at 2θ = 19.48°, which corresponds to the semicrystalline nature of PVA. This peak disappears completely after calcination, indicating the thermal decomposition and carbonization of the PVA matrix. For all calcined samples electrospun for 10, 20, and 30 min, distinct diffraction peaks are observed at 2θ = 43.32° and 51.82°, corresponding to the (111) and (200) planes, respectively. These peaks match well with the standard JCPDS card no. 004-0850, confirming the presence of metallic nickel (Ni^0^). The formation of Ni^0^ can be attributed to the partial reduction of NiO nanoparticles by the carbon derived from PVA during the calcination process in an inert or low-oxygen atmosphere. The consistent appearance of these peaks across all 3 samples suggests that electrospinning duration does not significantly affect the crystalline phase composition of the resulting NiO/carbon nanofibers.

##### FTIR analysis of nanofibers before and after calcination

**Figure 5:**
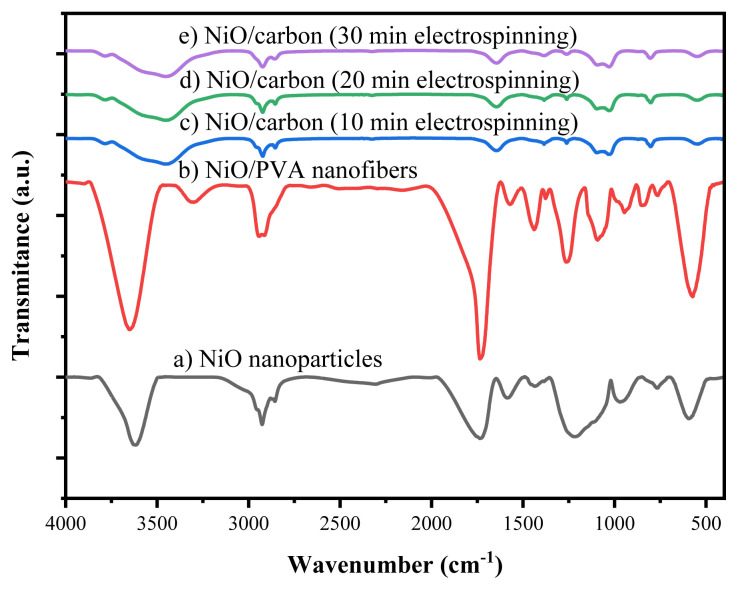
FTIR spectra of NiO nanomaterials, NiO/PVA nanofibers, and NiO/carbon nanofibers electrospun for 10, 20, and 30 min.

Figure 5 presents the FTIR spectra of NiO nanoparticles, NiO/PVA nanofibers before calcination, and NiO/carbon nanofibers electrospun for 10, 20, and 30 min after calcination at 500 °C. A characteristic peak at 577 cm^−1^ corresponds to Ni–O vibrations, confirming the presence of NiO nanocrystals. Absorption bands in the range of 750–1500 cm^−1^ are attributed to adsorbed CO_2_, with a peak at 1240 cm^−1^ indicating the presence of CO_3_^2−^ species. The band at 1727 cm^−1^ is associated with H–O–H bending vibrations from atmospheric moisture. In the uncalcined NiO/PVA nanofibers, broad absorption bands at 2930 cm^−1^ and 3622 cm^−1^ were observed, corresponding to C–H and –OH stretching vibrations, respectively. Notably, the broad –OH peak around 3310 cm^−1^ was not present in the calcined samples, confirming the thermal decomposition of the PVA component. The FTIR spectra of the calcined nanofibers obtained at different electrospinning times (10, 20, and 30 min) show no notable differences, indicating that electrospinning duration does not substantially affect the chemical structure of the resulting NiO/carbon nanofibers.

##### Raman analysis of nanofibers before and after calcination

**Figure 6:**
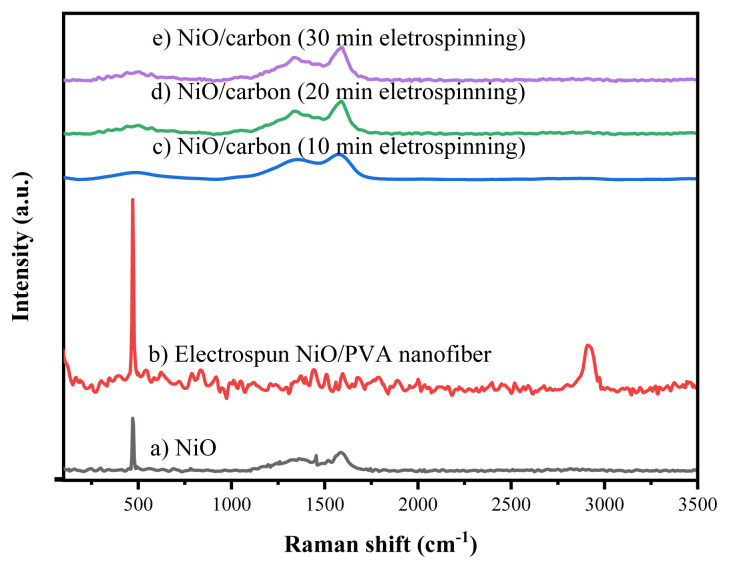
Raman spectra of NiO nanomaterials, NiO/PVA nanofibers, and NiO/carbon nanofibers electrospun for 10, 20, and 30 min.

Figure 6 presents the Raman spectra of NiO nanomaterials, NiO/PVA nanofibers, and NiO/carbon nanofibers electrospun for 10, 20, and 30 min. The NiO nanomaterials displayed characteristic Raman peaks at 470, 1340, and 1587 cm^−1^ that were also observed in both the as-spun and calcined nanofiber samples, confirming the presence and retention of NiO nanoparticles throughout the fabrication process. In the uncalcined NiO/PVA nanofibers, a notable peak is present at 2914 cm^−1^, corresponding to C–H stretching vibrations from the PVA matrix. This peak was not present after calcination at 500 °C, indicating the thermal decomposition of PVA. The Raman spectra of the calcined samples obtained at different electrospinning durations (10, 20, and 30 min) showed no significant spectral differences, suggesting that spinning time does not influence the fundamental structure of the NiO or carbon phases in the nanofibers.

##### TGA and differential thermogravimetric (DTG analysis

**Figure 7:**
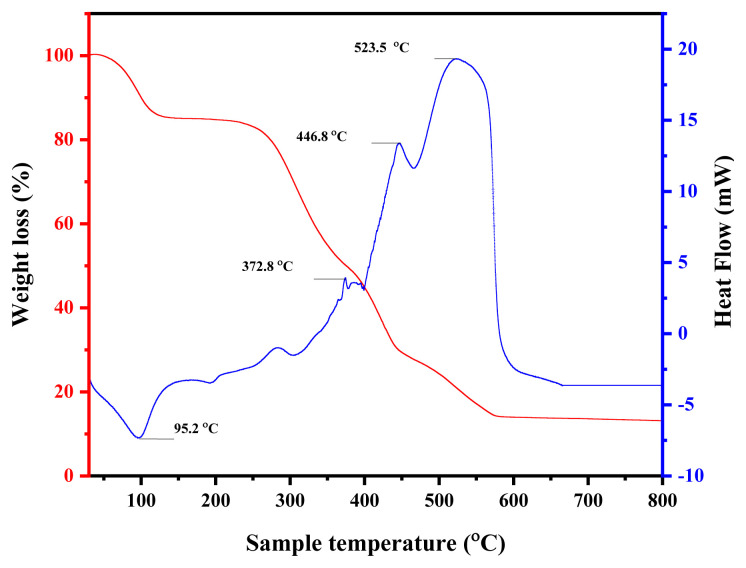
TGA curve of electrospun NiO/PVA nanofibers.

TGA and differential DTG analyses were carried out to investigate the thermal behavior of electrospun NiO/PVA nanofibers in a nitrogen atmosphere from 30 °C to 800 °C, as shown in Figure 7. The decomposition proceeded in 5 distinct stages. The first stage (30–140 °C) corresponded to the removal of physically adsorbed water, resulting in a mass loss of approximately 15%. Between 140 °C and 450 °C, the decomposition of PVA occurred via dehydration and cleavage of polymer chains, contributing to a major weight loss of about 57%. A glass transition was observed at 196 °C, followed by more severe degradation processes near 467 °C and 523 °C, where polymer scission and breakdown of carbon backbones occurred, resulting in a further 13% weight reduction. Beyond 600 °C, the TGA curve flattened, indicating no significant mass loss and thermally stable NiO and minor residual carbon or reduced nickel in the remaining residue. The final residual weight (14%) aligns well with the expected content of NiO after complete degradation of the PVA matrix.

This TGA/DTG profile not only confirms the thermal decomposition pathway of the NiO/PVA precursor but also indirectly captures the transformation into NiO/carbon nanofibers and ultimately NiO. Therefore, separate TGA analyses for the NiO/carbon nanofibers were not performed, as their thermal history and final composition are already reflected in the original TGA curve of the precursor. Likewise, pure NiO nanoparticles were not analyzed due to their well-documented thermal stability and negligible mass change under inert conditions.

##### PL analysis

**Figure 8:**
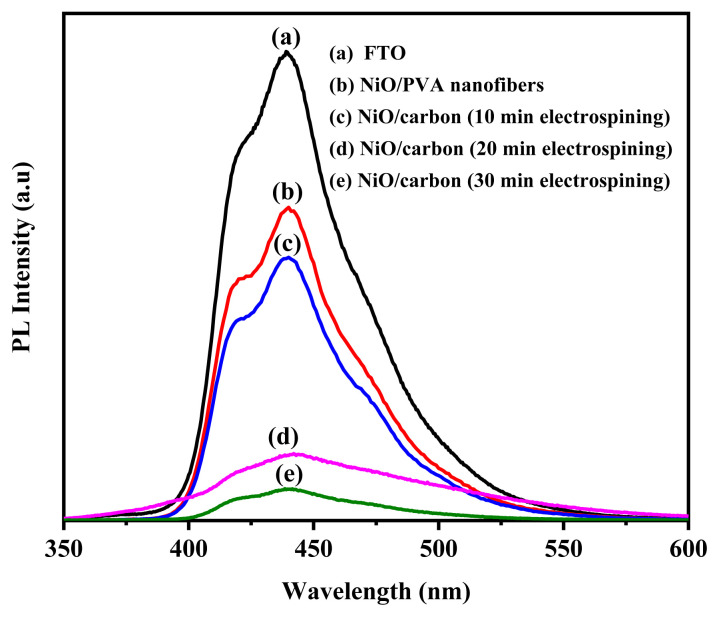
Fluorescence emission spectra of FTO glass, NiO/PVA nanofibers before heating, and fibers after heating at different electrospinning times.

Figure 8 presents the PL spectra of FTO glass, NiO/PVA fibers before calcination, and NiO/carbon nanofibers prepared with different electrospinning durations. A characteristic emission peak around 440 nm was observed in all samples, where the FTO glass had the highest intensity. As the processing time increased, the PL intensity gradually decreased, with the lowest emission recorded for the NiO/carbon nanofibers electrospun for 30 min. This reduction in PL intensity suggests a decrease in electron-hole recombination and fewer defect states in the NiO/carbon structure, indicating enhanced charge transport properties. These results show the potential of NiO/carbon nanofibers, particularly those with longer electrospinning times, as efficient HTLs in PSCs.

### 3.2. The solar energy conversion efficiency

**Figure 9:**
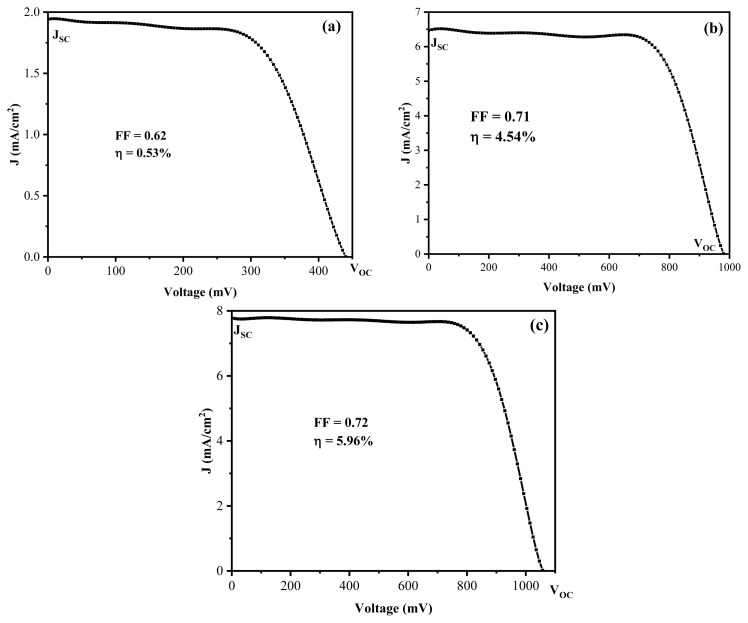
Characteristic J–V curves of solar cells according to model 1 (a), model 2 (b), and model 3 (c).

The performance of the solar cell models with varying hole materials, denoted as models 1, 2, and 3, was evaluated by measuring the current density (J) and voltage (V), as illustrated in Figure 9. The solar energy conversion efficiency is defined as the ratio of the maximum output power to the incident power of the light source. The photoelectric conversion efficiency, represented as a percentage, is calculated using the formula:


(1) $$\eta \;(\%)=\frac{{\text{V}}_{oc}*{\text{J}}_{\text{sc}}*\text{FF}}{{\text{P}}_{\text{in}}}$$

where V_oc_ is the open-circuit voltage, J_sc_ is the short-circuit current density, FF is the fill factor, and P_in_ is the incident optical power (100 mW/cm^2^).

Table presents the basic parameters of the 3 solar cell models, including open-circuit voltage (V_oc_), short-circuit current (I_sc_), current density (J_sc_), fill factor (FF), and efficiency (ɳ). The efficiency values for models 1, 2, and 3 were 0.53%, 4.54%, and 5.96%, respectively.

The performance of PSCs is strongly influenced by the structure and functionality of the HTL, particularly in terms of its composition, morphology, and thickness. In this study, NiO/carbon nanofiber HTLs were fabricated by electrospinning NiO/PVA nanofibers followed by calcination. The influence of electrospinning duration on device efficiency was systematically evaluated.

When the electrospinning time was limited to 10 min, the resulting NiO/PVA nanofiber layer was too thin to form a continuous and uniform HTL across the FTO substrate. Consequently, the device achieved a low power conversion efficiency (PCE) of 0.53%, likely due to incomplete coverage, inefficient hole extraction, and elevated interfacial recombination losses. By increasing the electrospinning time to 20 min, the device had a substantial performance improvement, reaching a PCE of 4.54%. This more than 8-fold enhancement can be attributed to the formation of a more interconnected and porous NiO/carbon nanofiber network that provides better interfacial contact with the perovskite layer. The combination of p-type NiO and conductive carbon residues significantly facilitates hole extraction and transport while minimizing charge recombination at the interface. Extending the electrospinning time to 30 min resulted in a further increase in PCE to 5.96%. However, the improvement was less pronounced compared to the previous step. Although a thicker HTL enhances electrical conductivity and suppresses recombination more effectively, it may also introduce longer charge transport pathways or increase internal resistance that could slightly compromise overall performance. In summary, the electrospun NiO/carbon nanofiber HTL plays a critical role in improving the electrical properties of PSCs through enhanced hole mobility, favorable energy level alignment, and suppression of charge recombination. However, these benefits are highly dependent on achieving an optimal thickness. Excessively thin or thick layers both lead to performance trade-offs. Therefore, fine-tuning the electrospinning duration is essential to balance conductivity, coverage, and charge extraction efficiency, ultimately maximizing PSC performance.

## 4. Conclusion

This study shows the potential of electrospun NiO/carbon nanofibers as an effective HTL in PSCs. By incorporating NiO nanoparticles into electrospun PVA nanofibers and converting them through carbonization, we developed a hybrid HTL that combines the favorable hole extraction properties of NiO with the conductivity and stability of carbon. Device performance tests showed a clear correlation between electrospinning duration and solar cell efficiency, with the optimal processing time yielding a PCE of 5.96%. These results highlight the importance of tuning fabrication parameters, such as NiO content and HTL thickness, to enhance charge transport and reduce recombination losses. The successful integration of NiO/carbon nanofibers offers a promising, low-cost strategy for improving PSC performance, paving the way for more stable and scalable perovskite-based photovoltaic technologies.

## Figures and Tables

**Table:** Fundamental parameters of the 3 solar cell models.

Model	V_oc_ (V)	I_sc_ (mA)	J_sc_ (mA/cm^2^)	FF	ɳ (%)
(1)	0.440	1.74	1.94	0.62	0.53
(2)	0.988	5.82	6.47	0.71	4.54
(3)	1.057	7.00	7.78	0.72	5.96

## References

[1] Van Pham DT Mai NTN Quyen TTB Ngoc NH Long TVB Dye-sensitized solar cells based on electrospun Ag-doped TiO_2_/PVA nanofibers Iranian Journal of Chemistry and Chemical Engineering 2023 42 5 1398 1408 10.30492/ijcce.2022.551404.5370

[2] Noman M Khan Z Jan ST A comprehensive review on the advancements and challenges in perovskite solar cell technology RSC Advances 2024 14 8 5085 5131 38332783 10.1039/d3ra07518d PMC10851055

[3] Van Pham DT Phat VV Hoa NTQ Ngoc NH Ngan DTT Fabrication of electrospun BaTiO_3_/chitosan/PVA nanofibers and application for dye-sensitized solar cells IOP Conference Series: Earth and Environmental Science 2021 947 1 012017 10.1088/1755-1315/947/1/012017

[4] Burschka J Pellet N Moon SJ Humphry Baker R Gao P Sequential deposition as a route to high-performance perovskite-sensitized solar cells Nature 2013 499 7458 316 319 10.1038/nature12340 23842493

[5] Liu M Johnston MB Snaith HJ Efficient planar heterojunction perovskite solar cells by vapour deposition Nature 2013 501 7467 395 398 10.1038/nature12509 24025775

[6] Fatima Q Haidry AA Zhang H El Jery A Aldrdery M A critical review on advancement and challenges in using TiO_2_ as electron transport layer for perovskite solar cell Materials Today Sustainability 2024 27 100857 10.1016/j.mtsust.2024.100857

[7] Njema GG Kibet JK Ngari SM A review of interface engineering characteristics for high performance perovskite solar cells Measurement: Energy 2024 2 100005 10.1016/j.meaene.2024.100005

[8] Chen L Li X Zhang N Yu L Liu Z Non-ionic polymeric polyacrylamide (PAM) modified SnO_2_ electron transport layer for high-efficiency perovskite solar cells Solar Energy Materials and Solar Cells 2024 272 112907 10.1016/j.solmat.2024.112907

[9] Park NG Research direction toward scalable, stable, and high efficiency perovskite solar cells Advanced Energy Materials 2020 10 13 1903106 10.1002/aenm.201903106

[10] Yousaf S Zulfiqar S Shahi MN Warsi MF Al-Khalli NF Tuning the structural, optical and electrical properties of NiO nanoparticles prepared by wet chemical route Ceramics International 2020 46 3 3750 3758 10.1016/j.ceramint.2019.10.097

[11] Zhang A Li M Dong C Ye W Zhu Y Role of NiO in wide-bandgap perovskite solar cells based on self-assembled monolayers Chemical Engineering Journal 2024 494 153253 10.1016/j.cej.2024.153253

[12] Luo G Zhang Y Zhu Q An Z Lv P Ruthenium complex optimized contact interfaces of NiO nanocrystals for efficient and stable perovskite solar cells Solar RRL 2024 8 4 2300890 10.1002/solr.202300890

[13] Jiang S Wang R Li M Yu R Wang F Synergistic electrical and light management enables efficient monolithic inorganic perovskite/organic tandem solar cells with over 24% efficiency Energy & Environmental Science 2024 17 1 219 226

[14] Su G Yu R Dong Y He Z Zhang Y Crystallization Regulation and Defect Passivation for Efficient Inverted Wide-Bandgap Perovskite Solar Cells with over 21% Efficiency Advanced Energy Materials 2024 14 4 2303344 10.1002/aenm.202303344

[15] Li MH Gong X Wang S Li L Fu J Facile hydrogen bonding assisted crystallization modulation for large area high-quality CsPbI_2_Br films and efficient solar cells Angewandte Chemie 2024 136 10 e202318591 10.1002/ange.202318591 38230583

[16] Nandi P Park H Shin S Lee JW Kim JY NiO as hole transporting layer for inverted perovskite solar cells: a study of x-ray photoelectron spectroscopy Advanced Materials Interfaces 2024 11 8 2300751 10.1002/admi.202300751

[17] Mann DS Kwon SN Thakur S Patil P Jeong KU Suppressing redox reactions at the perovskite-nickel oxide interface with zinc nitride to improve the performance of perovskite solar cells Small 2024 20 24 2311362 10.1002/smll.202311362 38192000

[18] Nkele AC Nwanya AC Nwankwo NU Osuji RU Ekwealor ABC Investigating the properties of nano nest-like nickel oxide and the NiO/Perovskite for potential application as a hole transport material Advances in Natural Sciences: Nanoscience and Nanotechnology 2019 10 4 045009 10.1088/2043-6254/ab5102

[19] Sagadevan S Podder J Investigations on structural, optical, morphological and electrical properties of nickel oxide nanoparticles International Journal of Nanoparticles 2015 8 3–4 289 301 10.1504/ijnp.2015.073731

[20] Manikandan A Judith Vijaya J John Kennedy L Comparative investigation of NiO nano- and microstructures for structural, optical and magnetic properties Physica E: Low-dimensional Systems and Nanostructures 2013 49 117 123 10.1016/j.physe.2013.02.013

[21] Golshani Z Arjmand F Maghsoudi S Hosseini SMA Fe_2_O_3_–NiO doped carbon counter electrode for high-performance and long-term stable photovoltaic perovskite solar cells Journal of Materials Research and Technology 2023 23 2612 2625 10.1016/j.jmrt.2023.01.178

[22] Zou Y Cao F Chen P He R Tong A Stable and highly efficient all-inorganic CsPbBr_3_ perovskite solar cells by interface engineering with NiO NCs modification Electrochimica Acta 2022 435 141392 10.1016/j.electacta.2022.141392

[23] Mariani P Najafi L Bianca G Zappia MI Gabatel L Low-temperature graphene-based paste for large-area carbon perovskite solar cells ACS Applied Materials & Interfaces 2021 13 19 22368 22380 10.1021/acsami.1c02626 33969983 PMC8289184

[24] Hu J Xiong X Guan W Long H Recent advances in carbon nanomaterial-optimized perovskite solar cells Materials Today Energy 2021 21 100769 10.1016/j.mtener.2021.100769

[25] Chen D Xie Y Chen T Zhang T Huang Y Low-temperature water-processed NiO hole transport layers for high-efficiency CH_3_NH_3_PbI_3_ perovskite solar cells Optical Materials 2024 149 114997 10.1016/j.optmat.2024.114997

[26] Meng F Wang D Chang J Li J Wang G Application of carbon materials in conductive electrodes for perovskite solar cells Solar RRL 2024 8 6 2301030 10.1002/solr.202301030

[27] Wang X Gao Y Ma J Guo J Zeng Y Back interface engineering by 2D layered N–Ti_3_C_2_ in low-cost carbon based all-inorganic hole transport layer free perovskite solar cells Solar Energy Materials and Solar Cells 2024 266 112687 10.1016/j.solmat.2023.112687

[28] Prajongtat P Sriprachuabwong C Wongkanya R Dechtrirat D Sudchanham J Moisture-resistant electrospun polymer membranes for efficient and stable fully printable perovskite solar cells prepared in humid air ACS Applied Materials & Interfaces 2019 11 31 27677 27685 10.1021/acsami.9b05032 31305061

[29] Sun Q Zhou S Shi X Wang X Gao L Efficiency enhancement of perovskite solar cells via electrospun CuO nanowires as buffer layers ACS Applied Materials & Interfaces 2018 10 13 11289 11296 10.1021/acsami.7b19335 29542316

[30] Sarkar P Tripathy SK Baishnab KL Polyvinylpyrrolidone capped electrospun CH_3_NH_3_PbCl_3_ perovskite film as the electron transport layer in perovskite solar cell application Solar Energy 2021 230 390 400 10.1016/j.solener.2021.10.053

[31] Shekaryar H Norouzbahari S A review on versatile applications of polyvinyl alcohol thin films, specifically as sensor devices Polymer Engineering & Science 2024 64 2 455 468 10.1002/pen.26569

[32] Van Pham DT Nhi PTY Long TVB Nguyen CN Nhan LM Electrospun Fe-doped TiO_2_/chitosan/PVA nanofibers: Preparation and study on photocatalytic and adsorption properties Materials Letters 2022 326 132930 10.1016/j.matlet.2022.132930

[33] Cai C Zhou K Guo H Pei Y Hu Z Enhanced hole extraction by NiO nanoparticles in carbon-based perovskite solar cells Electrochimica Acta 2019 312 100 108 10.1016/j.electacta.2019.04.191

[34] Upadhyaya A Negi CMS Yadav A Gupta SK Verma AS Synthesis and characterization of methylammonium lead iodide perovskite and its application in planar hetero-junction devices Semiconductor Science and Technology 2018 33 6 065012 10.1088/1361-6641/aac066

